# Macrophage Metabolism As Therapeutic Target for Cancer, Atherosclerosis, and Obesity

**DOI:** 10.3389/fimmu.2017.00289

**Published:** 2017-03-15

**Authors:** Xenia Geeraerts, Evangelia Bolli, Sarah-Maria Fendt, Jo A. Van Ginderachter

**Affiliations:** ^1^Laboratory of Myeloid Cell Immunology, VIB Inflammation Research Center, VIB, Ghent, Belgium; ^2^Laboratory of Cellular and Molecular Immunology, Vrije Universiteit Brussel, Brussels, Belgium; ^3^Laboratory of Cellular Metabolism and Metabolic Regulation, VIB Center for Cancer Biology, VIB, Leuven, Belgium; ^4^Laboratory of Cellular Metabolism and Metabolic Regulation, Department of Oncology, KU Leuven and Leuven Cancer Institute (LKI), Leuven, Belgium

**Keywords:** M1–M2 macrophage polarization, tumor-associated macrophages, microenvironment, immunometabolism, metabolic reprogramming, metabolic therapy, cancer, inflammatory diseases

## Abstract

Macrophages are not only essential components of innate immunity that contribute to host defense against infections, but also tumor growth and the maintenance of tissue homeostasis. An important feature of macrophages is their plasticity and ability to adopt diverse activation states in response to their microenvironment and in line with their functional requirements. Recent immunometabolism studies have shown that alterations in the metabolic profile of macrophages shape their activation state and function. For instance, to fulfill their respective functions lipopolysaccharides-induced pro-inflammatory macrophages and interleukin-4 activated anti-inflammatory macrophages adopt a different metabolism. Thus, metabolic reprogramming of macrophages could become a therapeutic approach to treat diseases that have a high macrophage involvement, such as cancer. In the first part of this review, we will focus on the metabolic pathways altered in differentially activated macrophages and link their metabolic aspects to their pro- and anti-inflammatory phenotype. In the second part, we will discuss how macrophage metabolism is a promising target for therapeutic intervention in inflammatory diseases and cancer.

## Introduction

Immunometabolism is a fast evolving field, which determines the metabolic machinery of immune cells and investigates how changing their metabolic phenotype affects immune cell function. It is known that the microenvironment shapes the metabolism of cells, which in turn contributes to their functionality. Environmental signals involved in metabolic regulation are cytokines, growth factors, oxygen levels, and nutrient availability. There is a growing evidence that immune cells in a specific microenvironment, such as inflamed tissue or tumors, reprogram their metabolic phenotype to fulfill cellular needs, such as survival, growth, and proliferation, or to carry out specific effector functions, such as phagocytosis and cytokine production. By changing the metabolism of immune cells, in particular, macrophages, it will be possible to modulate their function, which would be useful in diseases with a high macrophage commitment. Hence, understanding immune cell metabolism and its regulation will be essential to use metabolism as a therapeutic target to affect disease outcome (1–3).

In this review, we provide an overview of the current knowledge concerning the metabolism of differentially activated macrophages. We will discuss recent findings on macrophage metabolism in the context of cancer and inflammatory diseases such as obesity and atherosclerosis. Furthermore, we will comment on promising metabolic targets for therapeutic purposes and approaches to reprogram macrophage metabolism in particular diseases.

## Overview on the Most Relevant Metabolic Pathways in Macrophages

Metabolism is a network of highly interconnected biochemical reactions required to generate metabolic products, such as energy and macromolecules from nutrients, provided by the microenvironment. Despite the plasticity of the metabolic network, immune cells, including macrophages, usually stick to a unique metabolic phenotype to accomplish their function. For example, while multiple ways [i.e., glycolysis and oxidative phosphorylation (OXPHOS)] exist to produce energy, macrophages generally prefer particular pathways for energy production in relation to their functional requirements.

A pathway that allows energy and biomass production is glycolysis. During glycolysis, extracellular glucose is taken up by the cell and subsequently converted to two molecules of pyruvate and ATP whereas NAD^+^ is converted to NADH. To maintain glycolytic flux, cells can regenerate NAD^+^ by converting pyruvate into lactate. Glycolysis also provides the intermediate glucose-6-phosphate, which is the first molecule of the pentose phosphate pathway (PPP). The PPP consists of an oxidative branch that produces NADPH that is required to maintain the cellular redox balance and the production of fatty acids. The nonoxidative PPP branch provides intermediates used as precursors for nucleotide and amino acid synthesis. Although glycolysis is not the most efficient way to generate high amounts of ATP (two ATP molecules per glucose molecule), high glycolytic rates allow the cell to quickly produce sufficient energy and biosynthetic intermediates for cell growth and to fulfill its functional demands. For instance, the *in vivo* functions of macrophages not only encompass the insurance of tissue homeostasis under steady state but also a multitude of activities such as phagocytosis and cytokine production, upon activation (4). These functions have been referred to as SHIP: sample, heal, inhibit, and present (antigen) (5). Other cells that use glycolysis as major pathway for biomass production are cancer cells (6). Already in the early twentieth century, Otto Warburg postulated that cancer cells preferentially convert glucose into lactate, even in the presence of oxygen, a process better known as the Warburg effect (7). On the other hand, in the presence of oxygen, cells usually produce ATP via the electron transport chain (ETC) which is coupled to the tricarboxylic acid (TCA) cycle. Acetyl coenzyme A (acetyl-CoA) usually serves as the entry point of glycolytic carbon into the TCA cycle. The reducing equivalents NADH and FADH_2_, generated by the TCA cycle, serve as electron carriers that transfer electrons through ETC for OXPHOS, an oxygen-driven process that produces high amounts of ATP (theoretically up to 36 ATP molecules per glucose molecule). Moreover, cells can use different carbon sources, such as glutamine or fatty acids, to fuel into the TCA cycle. While glutamine can be converted into the TCA cycle intermediate α-ketoglutarate, the fatty acid oxidation pathway (FAO, also known as β-oxidation) degrades fatty acids into acetyl-CoA, NADH, and FADH_2_, which are further used to produce ATP. In general, OXPHOS is a highly efficient way for ATP production, preferred by cells with high energy demands or cells that require longevity to function over a long period of time (3, 8).

Besides energy production, intermediates from different metabolic pathways, such as glycolysis, PPP, and the TCA cycle can be used as precursors for *de novo* synthesis of nucleotides, fatty acids, and amino acids, which are essential building blocks for the cell. This requires increased replenishment of metabolic pathway intermediates via anaplerotic reactions. A well-known example is the replenishment of TCA cycle intermediates via direct conversion of pyruvate into oxaloacetate (OAA) by pyruvate carboxylase, production of α-ketoglutarate from glutamate or the conversion of adenylosuccinate into fumarate.

## Metabolic Signature of Macrophages

### Macrophage Activation States

Macrophages are considered as polyvalent cells in our body, playing a key role during embryonic development and contributing to tissue repair and inflammation (9). As a consequence, macrophages have a high plasticity and are able to adapt their phenotype, as instructed by their microenvironment and in agreement with their functional requirements (10). This has resulted in a spectrum model of macrophage activation, illustrating the divergent transcriptome of macrophages exposed to a broad variety of cues (11). Furthermore, this has led to a new proposal of nomenclature, whereby the triggers that determine the macrophage’s phenotype are specified (12). Additionally, other nomenclatures have been proposed to distinguish macrophage populations based on their function and encompassing both *in vitro* and *in vivo* situations (13). Despite the diversity of signals they can be subjected to and the different proposals for classifying them, macrophages for a long time have been classified in two main groups, representing the extremes of a continuum, namely “classically activated” or M1 and “alternatively activated” or M2 macrophages (14–16). Upon stimulation with interferon-γ (IFNγ) and toll-like receptor (TLR) ligands, such as lipopolysaccharides (LPS), macrophages obtain a pronounced pro-inflammatory M1 phenotype, characterized by the secretion of pro-inflammatory cytokines and reactive nitrogen and oxygen species. Furthermore, M1 macrophages possess bactericidal and antitumor activity. Conversely, the Th2 cytokines interleukin (IL)-4 and IL-13 polarize macrophages toward an anti-inflammatory M2 phenotype. Alternatively, activated macrophages are involved in tissue remodeling, immunosuppression, and show phagocytic and protumoral activity (12, 17). Although this dichotomous M1–M2 model is an oversimplification that only represents two extremes in a spectrum of macrophage activation states, it has been found that under pathological conditions, macrophages *in vivo* regularly mimic these two polarization states. Moreover, the M1/M2 nomenclature has been extensively used in multiple papers that are being referenced here, justifying the use of this nomenclature throughout this review. Imposed by their microenvironment, macrophages adapt their metabolic phenotype to fulfill their function in homeostasis and inflammation (18).

Briefly, the metabolism of M1 macrophages is characterized by enhanced glycolysis, flux through the PPP, fatty acid synthesis, and a truncated TCA cycle, leading to accumulation of succinate and citrate. The metabolic profile of M2 macrophages is defined by OXPHOS, FAO, a decreased glycolysis, and PPP (19). In the next section, we will discuss in more detail the metabolic signature of M1 and M2 macrophages and focus on the link between metabolism and macrophage functionality.

### M1 Macrophage Metabolism Is Characterized by Aerobic Glycolysis, Fatty Acid Synthesis, and a Truncated TCA Cycle

**Figure 1:**
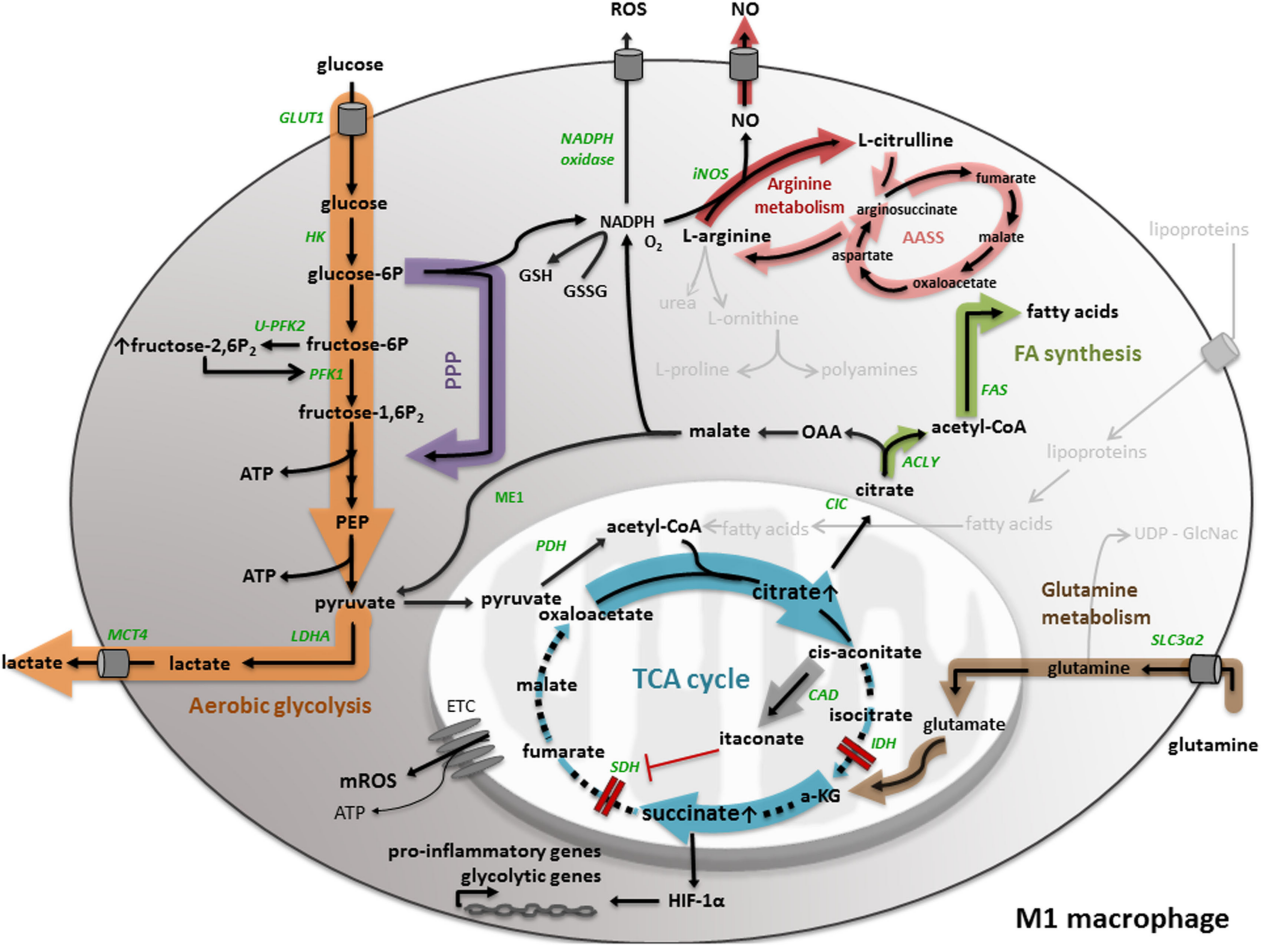
**M1 macrophage metabolism**. M1 macrophage metabolism is characterized by enhanced aerobic glycolysis, converting glucose into lactate. M1 macrophages have an increased flux through the pentose phosphate pathway (PPP), generating NADPH, used for the generation of the anti-oxidant glutathione (GSH) and the inflammatory mediators nitric oxide (NO) and reactive oxygen species (ROS). In M1 macrophages, the tricarboxylic acid (TCA) cycle is broken in two places, leading to the accumulation of succinate and citrate. While the accumulation of succinate leads to HIF-1α stabilization and the transcription of pro-inflammatory and glycolytic genes, citrate is used for the generation of fatty acids, NO, ROS, and the synthesis of itaconate. Another aspect of M1 macrophage metabolism is the conversion of l-arginine to NO and l-citrulline. All important metabolic reactions present in M1/M2 macrophages are shown in black, reactions shown in gray are absent or less pronounced. The metabolic pathways strongly upregulated by M1/M2 macrophage polarization are highlighted by a colored shadow, the width of the shadow illustrates the weight of a particular pathway in the macrophage activation state. All metabolic enzymes are indicated in green. Dotted lines represent impaired metabolic reactions. Abbreviations: α-KG: α-ketoglutarate; AASS: aspartate–arginosuccinate shunt pathway; ACLY: ATP-citrate lyase; CAD: *cis*-aconitate decarboxylase; CIC: mitochondrial citrate carrier; ETC: electron transport chain; FAS: fatty acid synthase; GLUT: glucose transporter; HK: hexokinase; IDH: isocitrate dehydrogenase; iNOS: inducible nitric oxide synthase; LDH: lactate dehydrogenase; MCT: monocarboxylate transporter; ME: malic enzyme; OAA: oxaloacetate; PEP: phosphoenolpyruvate; PDH: pyruvate dehydrogenase; PFK: phosphofructokinase; SDH: succinate dehydrogenase; SLC: solute carrier.

Integrated transcriptional and metabolic network analysis revealed that activation of macrophages by IFNγ and LPS gives rise to a TCA cycle that is truncated at the level of isocitrate dehydrogenase (IDH) and succinate dehydrogenase (SDH) leading to the accumulation of succinate and citrate metabolites (Figure 1). The build-up of citrate is the result of transcriptional downregulation of IDH1, the enzyme responsible for the conversion of isocitrate (an isomer of citrate) into α-ketoglutarate. The accumulated citrate serves as a precursor for the synthesis of the macrophage-specific metabolite itaconic acid, which is a major feature of IFNγ/LPS-polarized macrophages. Further supporting this phenomenon, Jha and colleagues found that immunoresponsive gene 1 (irg1) is one of the most upregulated genes in IFNγ/LPS-treated macrophages. Irg1 codes for the enzyme *cis*-aconitate decarboxylase that converts aconitate (derived from citrate) to itaconic acid (20). To ensure carbon entry into the truncated TCA cycle, LPS-activated macrophages repress pyruvate dehydrogenase kinase 1, which in turn leads to sustained conversion of pyruvate into acetyl-CoA via pyruvate dehydrogenase (21).

A study in murine and human macrophages revealed an antimicrobial effect of itaconic acid against *Mycobacterium tuberculosis* and *Salmonella enterica*, most likely by inhibition of the bacterial glyxoylate shunt pathway enzyme isocitrate lyase. Hence, itaconic acid is a first example of how cellular metabolism is linked to the antimicrobial function of pro-inflammatory macrophages (22). Recently, itaconic acid has been put forward as a specific driver for succinate accumulation in LPS-stimulated macrophages (23, 24), since it inhibits SDH (Figure 1). Hence, itaconic acid explains the second truncation in the TCA cycle of IFNγ/LPS-treated macrophages. In accordance, it was shown that LPS-induced bone marrow-derived macrophages from irg1^−/−^ mice did not display succinate accumulation because of impaired itaconic acid production (23). Different mechanisms have been proposed to explain carbon flow to succinate within a dysfunctional TCA cycle. One of them is glutamine-dependent anaplerosis through α-ketoglutarate or glutamine-derived succinate replenishment via the GABA shunt pathway. Either way, LPS-stimulated macrophages showed high levels of the glutamine transporter Slc3a2 (25).

Metabolite concentration changes can directly alter the activity of signaling pathways (26). The accumulation of succinate in LPS-stimulated macrophages is an example of this regulation via metabolite concentrations, since it stabilizes HIF-1α by limiting prolyl hydroxylase domain activity (Figure 1). In turn, HIF-1α stabilization induces the expression of the pro-inflammatory cytokine IL-1β (25). Additionally, a recent study proposes that succinate may indirectly stabilize HIF-1α via the induction of reactive oxygen species (ROS) (27). In any case, mature IL-1β production requires inflammasome activation, which supports pro-IL-1β to IL-1β processing (4). Interestingly, also inflammasome activation may be regulated by metabolic cues, as demonstrated by the finding that mTORC1-induced hexokinase (HK)-1-dependent glycolysis activates the NLRP3 inflammasome in macrophages upon LPS activation (28).

Furthermore, the stabilization of HIF-1α by succinate can be linked to an increased glycolytic flux (Figure 1), which is another metabolic signature of classically activated M1 macrophages, considering that HIF induces several glycolytic genes, such as glucose transporter 1 (GLUT1) (29), PFKFB3 (30), and monocarboxylate transporter 4 (MCT4) (31). The fact that LPS is able to induce hypoxic gene expression in macrophages in the absence of hypoxia has been suggested before (32). The expression of GLUT1 in M1 macrophages emphasizes the importance of glucose as carbon source for pro-inflammatory macrophages. Freemerman and colleagues demonstrated that overexpression of GLUT1 in the murine macrophage cell line RAW264.7 induces an M1 phenotype, characterized by the expression of inflammatory mediators and the production of ROS (33). Another HIF-1α regulated gene is PFKFB3, which codes for the ubiquitous 6-phosphofructo-2-kinase/fructose-2,6-bisposphatase isoform (U-PFK2). The expression of U-PFK2 is considered as an underlying mechanism of a high glycolytic flux in M1 macrophages. In particular, upon IFNγ/LPS stimulation, the expression of PFK2 shifts from the liver isoform (L-PFK2) to the more active ubiquitous isoform (U-PFK2). U-PFK2 converts fructose-6-phosphate to fructose-2,6-biphosphate that in turn activates the glycolytic enzyme phosphofructo-1-kinase (PFK1) and consequently increases glycolytic flux (34, 35) because of its dominant kinase activity. Since the TCA cycle is truncated in M1 macrophages, lactate production regenerates the majority of the NAD^+^ needed to sustain glycolysis. Consequently, lactate is exported to the extracellular environment by (MCT4). Data indicate that knocking down MCT4 in LPS-induced macrophages leads to enhanced accumulation of intracellular lactate, a decrease in the expression of the glycolytic enzymes HK-2 and PFKFB3 and a reduction of the pro-inflammatory cytokines IL-6 and tumor necrosis factor (TNF)α (36).

Another aspect of M1 macrophages is an enhanced PPP (25), which is not unexpected considering the high glycolytic flux. The PPP generates NADPH, which is required as a cofactor for LPS-induced inducible nitric oxide synthase (iNOS) to catabolize arginine into nitric oxide (NO) and l-citrulline (Figure 1). To sustain antimicrobial NO production, imported l-citrulline can be recycled to arginine by the so-called aspartate–arginosuccinate shunt pathway (AASS) (37). NO is not only an antimicrobial agent, but is also put forward as a key regulator of M1 macrophage metabolism. NO actually causes nitrosylation of the iron–sulfur-containing ETC complexes and consequently inhibits mitochondrial respiration and OXPHOS (38, 39). Recent research by Van den Bossche and colleagues demonstrated that disturbed mitochondrial OXPHOS, caused by IFNγ/LPS-induced NO production, can prevent M1 to M2 polarization after IL-4 stimulation (40). Furthermore, PPP-produced NADPH can be used as a cofactor for NADPH oxidase, which is involved in the generation of ROS (41). In general, the NADPH-driven production of the inflammatory mediators NO and ROS once again indicates the tight link between M1 metabolism and its antimicrobial functionality. Nevertheless, TLR1/2/4 signaling in macrophages induces mitochondrial ROS production, despite increased NADPH production via the PPP, which also contributes to the bactericidal activity of macrophages (42).

An additional concept that is related to M1 macrophage metabolism is maintenance of the cellular redox balance. NADPH can be used to generate the anti-oxidant glutathione (Figure 1), which is essential to maintain redox homeostasis and prevent cellular damage by ROS (8). NAPDH can be produced by the PPP, and also citrate can be exported to the cytosol where it contributes to NADPH production and regulation of the redox balance. In particular, the mitochondrial citrate carrier (CIC), encoded by the Slc25a1 gene, exports citrate from the mitochondria to the cytosol where it is cleaved back to acetyl-CoA and OAA by the enzyme ATP-citrate lyase (ACLY). In macrophages, the expression of both CIC and ACLY is induced by inflammatory stimuli, such as LPS, TNFα, and IFNγ through NF-kB and/or signal transducer and activator of transcription (STAT) signaling (43–45). Within the cytosol, OAA can be reduced to malate, which is converted to pyruvate by malic enzyme (ME1) with production of NADPH. However, in their network analysis, Jha and colleagues did not detect ME1 activity in IFNγ/LPS-induced macrophages (20). Taken together, citrate-derived NADPH can be used, as described previously, for NO and ROS production and might support the redox balance (43–45), which again associates a metabolic intermediate to the pro-inflammatory functionality of M1 macrophages.

Moreover, it has been shown in several studies that specific metabolites, such as acetyl-CoA, can regulate the activation of chromatin-modifying enzymes, making the link between metabolism and epigenetics. For example, citrate-derived acetyl-CoA can be used for histone acetylation (46). Especially the expression of some glycolytic genes, such as HK-2, PFK1, and lactate dehydrogenase-A is regulated in an ACLY-dependent manner and thus subjected to histone acetylation (47). Besides, acetyl-CoA can be used for the biosynthesis of fatty acids (Figure 1), a last aspect of M1 macrophage metabolism we want to discuss. It was demonstrated before that LPS activation of TLR4 in macrophages induces lipid accumulation through several mechanisms, among which enhanced fatty acid uptake by the fatty acid transporter CD36, increased triglyceride synthesis, and diminished triglyceride lipolysis (48). Moreover, it was found that upon M-CSF mediated monocyte to macrophage differentiation, lipid synthesis increased through upregulation of the nuclear transcription factor sterol regulatory element-binding protein-1c, inducing expression of fatty acid synthesis-related genes, such as fatty acid synthase (49). Moreover, fatty acids are used as precursor for prostaglandin production in macrophages stimulated by TNFα, LPS, or IFNγ (43–45).

Since citrate is involved in several mechanisms that shape the pro-inflammatory phenotype of M1 macrophages, such as production of NO, ROS, NAPDH, itaconic acid, fatty acids, and histone acetylation, M1 macrophages developed mechanisms to ensure the preservation of high citrate levels. As described before, IFNγ/LPS-treated macrophages show an upregulation of the AASS pathway, which connects the NO and TCA cycle. This pathway not only sustains NO production, but also replenishes citrate in the broken TCA cycle by anaplerosis (20).

Taken together, the above-described metabolic mechanisms of M1 macrophages contribute in a specific manner to their pro-inflammatory phenotype, highlighting the link between macrophage metabolism and functionality.

### The Metabolic Signature of M2 Macrophages Is Characterized by FAO, and an Oxidative TCA Cycle

The metabolic phenotype of M2 macrophages shows significant differences with M1 macrophages, which is comprehensible regarding their differential function as anti-inflammatory component and mediator of tissue homeostasis (19).

**Figure 2:**
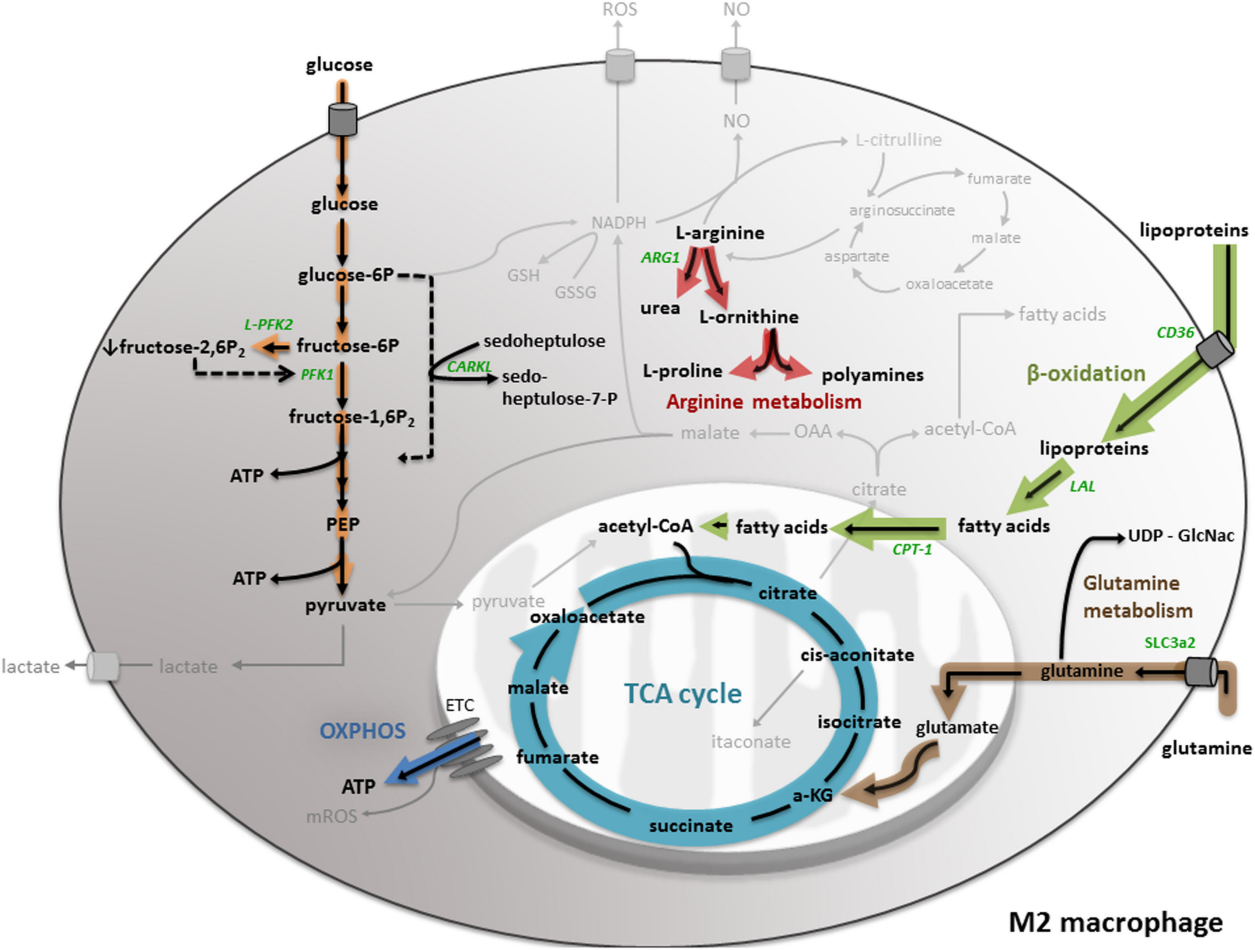
**M2 macrophage metabolism**. M2 macrophages mainly produce ATP through an oxidative TCA cycle coupled to oxidative phosphorylation (OXPHOS). To fuel the TCA cycle, M2 macrophages rely on fatty acid oxidation (or β-oxidation) and glutamine metabolism. Furthermore, M2 macrophages show a lowered glycolysis and pentose phosphate pathway (PPP). Moreover, M2 macrophages convert l-arginine into urea and l-ornithine, which serves as precursor for l-proline production. All important metabolic reactions present in M1/M2 macrophages are shown in black, reactions shown in gray are absent or less pronounced. The metabolic pathways strongly upregulated by M1/M2 macrophage polarization are highlighted in orange. All metabolic enzymes are indicated in green. Dotted lines represent impaired metabolic reactions. Abbreviations: α-KG: α-ketoglutarate; ARG: arginase; CD: cluster of differentiation; CPT: carnitine palmitoyl transferase; ETC: electron transport chain; LAL: lysosomal acid lipase; PEP: phosphoenolpyruvate; PFK: phosphofructokinase; SLC: solute carrier.

One of the major metabolic differences between M1 and M2 macrophages is their energy metabolism. While M1 macrophages preferentially obtain their energy from glycolysis, M2 macrophages mainly produce ATP through an oxidative TCA cycle coupled to OXPHOS (Figure 2) (50). To fuel an oxidative TCA cycle, IL-4-stimulated macrophages rely on FAO (also known as β-oxidation) (51) and glutamine metabolism (20). In IL-4-induced macrophages, the important sources of fatty acids are triacylglycerol-rich lipoproteins, such as LDL and VLDL. These are taken up by the scavenger receptor CD36 and catabolized in the lysosome by lysosomal acid lipase (LAL). Studies in CD36- and LAL-deficient mice indicated that CD36-mediated lysosomal lipolysis of lipoproteins is essential for proper M2 activation (52). Another study demonstrated that expression of a permanently active mutant of carnitine palmitoyl transferase (CPT)-1a, which is essential for the transport of long-chain fatty acids along the mitochondria, enhanced FAO in macrophages, and reduced inflammation (53). However, a recent study indicated that macrophage-specific CPT2 depletion inhibits FAO, while not impairing M2 polarization after IL-4 stimulation (54). These data suggest that CPT1 might have an extra function in M2 polarization, independent of FAO (4). As mentioned before, glutamine can fuel the TCA cycle via anaplerotic generation of α-ketoglutarate. Furthermore, glutamine contributes to UDP-GlcNAc synthesis via the hexosamine pathway. UDP-GlcNAc leads to protein glycosylation, including the M2 markers macrophage mannose receptor (MMR) and macrophage galactose binding lectin (Mgl1) (20).

In IL-4/IL-13-stimulated macrophages, the upregulation of FAO and mitochondrial biogenesis is orchestrated by the combined action of STAT6, peroxisome proliferator-activated receptors (PPARs), and PGC-1β (55). Stimulation with Th2 cytokines IL-4 and IL-13 induces a cytoplasmic signaling cascade, resulting in activation of the transcription factor STAT6, which in turn induces expression of PPARδ, PPARγ, and the coactivator protein PGC-1β (55, 56). Additionally, the expression of M2 markers, such as MMR, is regulated by the interplay of these components (57). Accordingly, the knockdown of PGC-1β was shown to decrease FAO upon IL-4 stimulation and impaired the suppression of pro-inflammatory cytokine production by IL-4, which underlines the importance of PGC-1β to sustain the anti-inflammatory phenotype of M2 macrophages (51).

Besides differences in the energy metabolism, M1 and M2 macrophages show an opposing arginine metabolism (Figure 2), which is correlated to their functional polarization. While M1 macrophages upregulate iNOS and metabolize l-arginine to the antimicrobial agent NO and l-citrulline, M2 macrophages catalyze l-arginine to urea and l-ornithine by inducing arginase (ARG-1). l-ornithine serves as precursor for l-proline production, which is used for collagen synthesis and accordingly contributes to wound repair, a key function of M2 macrophages (58). The expression of ARG-1 is induced by the Th2 cytokines IL-4 and IL-10 (59) via activation of the transcription factor STAT6 (60, 61). Since NO is no longer produced in M2 macrophages, it cannot block the ETC and subsequently enables OXPHOS (40), pointing out the strong interconnection within the metabolic network of M2 macrophages. A recent study by Ref. (62) stated that ornithine decarboxylase (ODC), involved in polyamine metabolism, directly regulates macrophage activation. The authors showed that ODC-deficiency in macrophages alters histone modifications and changes the chromatine structure, leading to up-regulated transcription of M1 genes and increased inflammation during bacterial infections with *Helicobacter pylori* and *Citrobacter rodentium* (62).

In contrast to M1 macrophages, M2 macrophages show a lowered glycolysis (Figure 2). IL-4/IL-13-stimulated macrophages express the L-PFK2 isoform, encoded by the PFKFB1 gene, which has a dominant biphosphatase activity, resulting in low levels of the glycolytic activator fructose-2,6-biphosphate (34). Correlated to low glycolytic rates, M2 macrophages have a limited flux through the PPP. IL-4/IL-13 stimulation promotes the expression of kinase-like protein (CARKL), a sedoheptulose kinase that catalyzes the production of sedoheptulose-7-phosphate and limits PPP flux in a not fully understood manner. Moreover, overexpression of CARKL in macrophages leads to decreased production of pro-inflammatory cytokines, which is consistent with the M2 phenotype (63).

## Macrophage Metabolism as a Promising Target for Therapeutic Intervention in Cancer and Other Inflammatory Diseases

In multiple inflammatory diseases, macrophages are leading cells affecting disease outcome. For example, a tumor is considered as a nonhomogeneous mass wherein a complex interaction exists between cancer cells and tumor-infiltrating immune cells, such as macrophages. These so-called tumor-associated macrophages (TAM) are one of the most prominent immune cells in a tumor. Although macrophages can target transformed cells, cancer cells evade this defense by reprogramming macrophages so that they support tumor progression (17, 64). The cancer context is a good example where metabolic targeting is already assessed as therapeutic target. Cancer cells have, as a consequence of their highly proliferative potential, a metabolic signature that is notably different compared to nonmalignant cells. Several drugs that specifically target the metabolic pathways of cancer cells are in clinical trials (65, 66). However, in other inflammatory diseases, such as obesity and atherosclerosis, macrophages are highly involved in disease progression. Hence, macrophages are an intriguing target for therapy in several disorders. In the next section, we will discuss recent discoveries concerning macrophage metabolism in cancer and inflammatory diseases and we will put forward interesting strategies for therapeutic intervention.

### Cancer: Repolarizing Macrophages toward a Pro-inflammatory Phenotype

**Figure 3:**
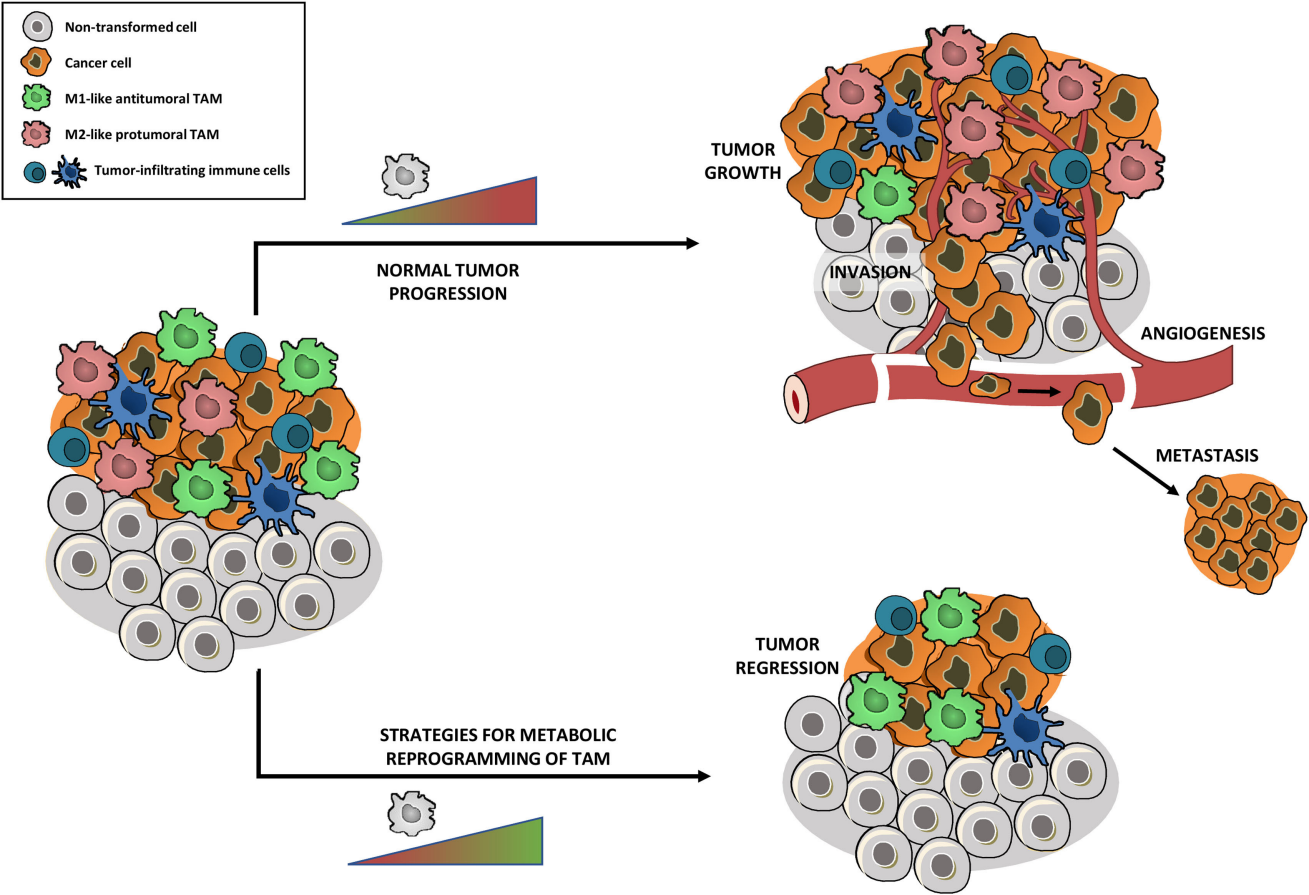
**Metabolic reprogramming of tumor-associated macrophages (TAM) toward an antitumoral phenotype might affect tumor growth**. Tumors are highly infiltrated by tumor-infiltrating immune cells, with TAM being amongst the most abundant ones. Within the same tumor, the co-existence of two distinct TAM subpopulations has been shown: M2-like protumoral TAM and M1-like antitumoral TAM. The TAM phenotype depends on the stage of tumor development, leading to a majority of M2-like TAM in late stage tumors which stimulate tumor growth, angiogenesis, invasion, metastasis, suppression of antitumor immunity, and mediation of therapy resistance. Strategies that metabolically reprogram protumoral M2 TAM into an antitumoral M1 phenotype, without depleting the full TAM population, could reduce tumor growth and metastasis and allow re-establishment of conventional cancer therapies.

Tumor-associated macrophages are amongst the most abundant inflammatory cells in tumors and a significant correlation was found between high TAM density and a worse prognosis for most cancers (67). By now, it is clear that TAM exert several tumor-promoting functions, including stimulation of angiogenesis, remodeling of the extracellular matrix, promotion of cancer cell survival, proliferation, invasion, extravasation and metastasis, and suppression of antitumor immunity (68). Within the same tumor, the co-existence of two distinct TAM subpopulations has been shown, both derived from tumor-infiltrating inflammatory monocytes: M2-like MHC-II^low^ TAM that reside in the hypoxic regions of the tumor and perform angiogenic, immunosuppressive and protumoral activities and M1-like MHC-II^high^ TAM that are present in the normoxic tumor regions and possess pro-inflammatory and antitumoral characteristics. Importantly, this dichotomous TAM phenotype remained valid in several independent transplantable and transgenic mouse tumor models (69–71). Studies indicate that the TAM phenotype depends on the stage of tumor development and that the majority of TAM in late stage tumors is M2-like (72) (Figure 3). M2-like TAM highly stimulate tumor progression and have been shown to affect the efficacy of anticancer treatments, contribute to therapy resistance, and mediate tumor relapse following conventional cancer therapy (9). Therefore, intervening with M2 TAM functionality is a plausible avenue for the development of new immunotherapies.

Before, TAM-targeted antitumor strategies were mainly based on the inhibition of macrophage recruitment to the tumor and suppression of TAM survival (73). For example, CCL2 blocking agents, which prevent monocyte recruitment to the tumor and subsequent generation of monocyte-derived TAM, have been shown to impair tumor progression in several tumor models (74–76). As a cautionary note, a recent study in breast carcinoma demonstrated that interruption of CCL2 inhibition was associated with increased cancer cell mobility and blood vessel formation, leading to accelerated metastasis and cancer death (77). Hence, ablation of TAM as a monotherapy might be insufficient for prolonged tumor control. Furthermore, general macrophage depletion could cause side effects, such as increased susceptibility for infections (78).

Therefore, strategies that reprogram protumoral M2 TAM into an antitumoral M1 phenotype, without depleting the full TAM population, gained attention and are currently highly investigated. In this context, Casazza and colleagues showed that genetic depletion of neuropilin-1 in TAM prevents the entry of TAM in the hypoxic regions of the tumor. TAM that accumulate in the normoxic tumor area adopt a more M1-like phenotype, which initiates a cascade of antitumor immunity, leading to reduced angiogenesis, tumor growth, and metastasis (79). Recent research indicated that blocking M-CSFR signaling impaired the differentiation of tumor-infiltrating monocytes into MHC-II^low^ M2-like TAM and consequently shifted the M1/M2 TAM balance toward the antitumoral TAM phenotype (71). Additionally, studies in glioblastoma illustrated that M-CSFR inhibition impaired the M2 phenotype and the tumor-promoting functions of TAM, without affecting TAM numbers (80).

Since macrophage metabolism is inextricably connected to its functionality, metabolic reprogramming of M2-like TAM might be an elegant way to repolarize TAM toward an antitumoral phenotype and thus affect tumor growth and metastasis. Although studies about TAM metabolism are rather limited at this moment, there is an emerging evidence that unraveling the TAM phenotype might lead to the identification of alternative, novel targets for TAM-directed intervention.

A study concerning TAM metabolism by Colegio and colleagues indicated that tumor-derived lactate is necessary to polarize TAM toward a protumoral M2 phenotype. Stimulation of bone marrow-derived macrophages with lactate was sufficient to induce the expression of the M2-related genes *Vegf, Arg1, Relma, Mgl1*, and *Mgl2*. Interestingly, stabilization of HIF-1α by tumor-derived lactate was the actual driving force for this M2 polarization. Moreover, by inducing *Vegf* and ARG-1, lactate fulfills a key role in shaping the protumoral phenotype of TAM. While *Vegf* induces angiogenesis, ARG-1 contributes to tumor growth by the generation of polyamines which act as a proliferative signal for mammalian cancer cells (81). This study proves that nutrients in the tumor microenvironment, such as lactate, can affect the phenotype of infiltrating immune cells, such as TAM.

Research by Penny and colleagues demonstrated that *in vitro* generated macrophages, cultured with tumor-conditioned media from a pancreatic ductal adanocarcinoma (PDAC) cell line, have a pronounced metabolic preference for aerobic glycolysis. In comparison to control macrophages, PDAC TAM showed enhanced angiogenesis and cancer cell extravasation, hence inducing metastasis. Treating PDAC TAM with 2-deoxyglucose, an inhibitor of the first glycolytic enzyme HK2, blocked the pro-metastatic TAM phenotype (82). This study links pancreatic TAM metabolism to aerobic glycolysis, which is in contrast to our current understanding of M2 macrophage metabolism, as extensively described before, claiming their lowered glycolysis and preference for oxidative mitochondrial metabolism.

A recent study by the group of Mazzone revealed that TAM metabolism directly affects tumor vasculature and metastasis, making the link between TAM metabolism and its protumoral functionality. REDD1, an inhibitor of mTOR, is highly expressed by TAM in the hypoxic regions of the tumor, which have been described previously as more M2-like macrophages with high angiogenic potential. Genetic deletion of REDD1 in hypoxic TAM induced mTOR activity, which in turn increased glucose uptake and directed hypoxic macrophage metabolism toward glycolysis. Enhanced glycolysis upon REDD1 deletion caused competition for glucose between hypoxic TAM and tumor endothelial cells. As a result of this competition, tumor vasculature is stabilized, thereby preventing metastasis (83). Thus, any approach diminishing REDD1 activity in TAM could be an interesting strategy to metabolically reprogram TAM toward a M1 phenotype. However, this should be combined with a therapy that allows to specifically target TAM, because REDD1 inhibition will stimulate mTOR signaling and glycolysis in cancer cells and boost their proliferation.

Hence, the challenge for next-level TAM-based antitumor therapies will be to identify metabolic targets that allow repolarizing TAM toward an antitumoral M1 phenotype (Figure 3), without boosting cancer cell metabolism. Therefore, strategies that are able to target TAM metabolism in a specific way will gain much attention. An even more interesting approach would be to look for metabolic targets that allow TAM repolarization and at the same time impair cancer cell metabolism. Therefore, drugs that have been shown to disturb cancer cell metabolism could be repurposed to metabolically shift TAM. In this context, drugs that inhibit FAO could be very promising. Several studies indicated the importance of FAO for different types of cancer cells (84). For instance, it has been shown that pharmacological inhibition of FAO impaired proliferation of human leukemia cells (85). On the other hand, blocking FAO in human glioblastoma cells induced oxidative stress and led to bioenergetic failure and cell death (86). A recent study by Schoors and colleagues demonstrated that FAO supports *de novo* nucleotide synthesis in endothelial cells and subsequently contributes to angiogenesis (87). As described before, FAO is related to the metabolism of IL-4/IL-13 induced M2 macrophages (51). Although it has not been shown that inhibiting FAO shifts M2-like TAM to a M1-like phenotype, impairing FAO could be a promising approach to target pro-tumoral macrophage, endothelial, and cancer cell metabolism at the same time.

The opportunity of targeting immune cell metabolism for cancer therapy is extensively studied in T cells. Potential strategies that aim to alter T-cell metabolism as an effective treatment against cancer recently evolved and have been reviewed in Ref. (88). Interestingly, it was shown that upon PD-1 ligation T-cell metabolism shifts from glycolysis to FAO, encouraging T-cell longevity and impairing their effector function. Hence, currently used immune checkpoint inhibitors, such as PD-1 blocking antibodies, could affect T-cell metabolism by altering glycolysis and subsequently enhance T-cell function (89). Besides immune cells, the metabolism of stromal cells, such as endothelial cells, gained much attention and highlighted interesting therapeutic strategies for cancer therapy (87, 90).

### Macrophage Polarization beyond Cancer: A Case Study on Obesity and Atherosclerosis

The concepts concerning macrophage polarization, as discussed above in the context of TAM, can be translated beyond cancer. Also for inflammatory diseases, such as atherosclerosis and obesity, metabolic repolarization of macrophages could be an interesting approach to affect disease outcome.

Atherosclerotic cardiovascular disease is a chronic inflammatory disorder, showing strong macrophage involvement. In case of hypercholesterolemia, apolipoprotein B-containing lipoproteins, such as LDL, accumulate in the arterial wall and are taken up by macrophages. These lipid-laden macrophages, better known as foam cells, lead to plaque formation and secrete inflammatory mediators that persist inflammation and promote plaque progression. Plaque rupture and subsequent formation of blood cloths form the basis for myocardial infarction and stroke, the leading causes of death in western countries (91, 92). Although both M1 and M2 macrophages are present in human atherosclerosis, M1 macrophages are the dominant phenotype linked to plaque progression (93). Several studies in mouse models for atherosclerosis demonstrated that inducing M1 polarization of macrophages enhanced disease progression. For example, deletion of the transcription factors NUR77 or KLF4, which both have been shown to drive M2 polarization, induced M1 polarization of macrophages, and enhanced atherosclerosis in apolipoprotein E^−/−^ mice (94, 95). In line with these studies, inducing M2 polarization through administration of the M2-related cytokine IL-13 reduced disease progression (96). Furthermore, lowering lipid levels or enhancing HDL levels in mouse models, induced atherosclerosis regression as a result of a switch toward M2 macrophages in plaques (97, 98). Hence, strategies that allow M2 polarization of macrophages could be beneficial in atherosclerosis disease. A recent study demonstrated that upregulating FAO in macrophages of hypercholesterolemic mice via miR-33 inhibition drives macrophages toward an M2 state and reduces atherosclerosis (99). This study emphasizes how metabolic reprogramming of macrophages can influence disease outcome.

Besides atherosclerosis, the relevance of interfering with macrophage metabolism gained much attention in the context of metabolic diseases, such as obesity. Low-grade systemic chronic inflammation leads to accumulation of macrophages in the adipose tissue of obese humans and mice. While adipose tissue macrophages (ATM) represent less than 10% of all adipose tissue cells in lean mice and humans, their percentage raises over 50% in extremely obese mice and near 40% in obese humans (100). Since macrophages are known as plastic cells that adapt their phenotype to the changing microenvironment, one could expect a switch in the phenotype of ATM during obesity, as described in Ref. (101). Macrophages in lean adipose tissue are considered as anti-inflammatory M2 macrophages, playing a role in maintaining adipose tissue homeostasis by cleaning cellular debris and lipid buffering (uptake and storage of lipids released by adipocytes) (102). Macrophages in obese adipose tissue have been described as pro-inflammatory M1 macrophages and are believed to be the major contributors of obesity-induced insulin resistance, leading to type-2 diabetes, due to the production of pro-inflammatory cytokines, such as TNF (103). More recent papers highlight the importance of the continuous exposure of ATM to lipids *in vivo*, leading to chronic lipid overloading. Xu et al. showed that ATM in obese adipose tissue are associated with increased liposomal biogenesis and lipid catabolism (104). More recently, Kratz and colleagues introduced the principle of metabolic activation of ATM, showing that treating macrophages with glucose, insulin, and fatty acids (palmitate) drives the pro-inflammatory ATM phenotype in obese mice (105). Hence, this study indicates that a metabolic trigger can lead to inflammatory macrophage activation, rather than a classic cytokine-driven activation. These new insights imply that interfering with metabolism is a promising approach to dampen the pro-inflammatory ATM phenotype and restore adipose tissue homeostasis in obesity.

Taken together, hyperactivated pro-inflammatory macrophages often contribute to disease progression in inflammatory disorders, such as atherosclerosis and obesity. Therefore, dampening their pro-inflammatory activity is a promising avenue to affect disease outcome. Although, similar to cancer, where repolarization of macrophages toward the pro-inflammatory phenotype has been established as beneficial in inflammatory diseases, such as obesity and atherosclerosis, it would be even more useful to do the opposite and reprogram macrophages toward an anti-inflammatory state. Especially in the case of atherosclerosis, forcing macrophages into an anti-inflammatory phenotype has been shown to induce atherosclerosis regression.

## Concluding Remarks

The growing interest and studies on immunometabolism, in particular, the metabolism of macrophages, raise new therapeutic opportunities to treat inflammatory diseases and cancer. In particular, the metabolic repolarization of macrophages seems to be an interesting approach to fight diseases that show a high macrophage involvement, such as cancer, obesity, and atherosclerosis. Nevertheless, there are some challenges for the future that need to be considered in the study of macrophage metabolism. One of them will be to either only selectively target macrophages or to find metabolic targets that do not affect the disease in a positive way, which is an existing risk in the case of cancer. On the other hand, macrophages should be considered as dynamic cells which adapt their phenotype and possibly also their metabolic state through the different phases of disease, which might hamper metabolic targeting. Furthermore, most of the macrophage metabolism studies have been done *in vitro*. However, during inflammation or tumor development, macrophages have to deal with a specific microenvironment, characterized by disturbed nutrient and oxygen availability, which instructs their metabolism and functionality. Hence, specialized experimental technologies, such as *in vivo* tracer analysis, will be unconditional to bring macrophage metabolism studies one step further to the *in vivo* level. We look forward to revolutionary discoveries in the area of macrophage metabolism that will lead to therapeutic targets that could affect disease outcome in inflammatory diseases and cancer.

## Author Contributions

XG has contributed in writing, reviewing, and editing the content of this article. EB has contributed in reviewing this article. S-MF and JVG have contributed in writing, reviewing, editing, conceptualization, and supervising this article. All authors have read and approved the manuscript.

## Conflict of Interest Statement

The authors declare that the research was conducted in the absence of any commercial or financial relationships that could be construed as a potential conflict of interest.
